# Methylation by Set9 modulates FoxO3 stability and transcriptional activity

**DOI:** 10.18632/aging.100471

**Published:** 2012-07-21

**Authors:** Daniel R. Calnan, Ashley E. Webb, Jamie L. White, Timothy R. Stowe, Tapasree Goswami, Xiaobing Shi, Alexsandra Espejo, Mark T. Bedford, Or Gozani, Steven P. Gygi, Anne Brunet

**Affiliations:** ^1^ Cancer Biology Program, Stanford University, Stanford, CA 94305; ^2^ Department of Genetics, Stanford University, Stanford, CA 94305; ^3^ Department of Biology, Stanford University, Stanford, CA 94305; ^4^ Department of Cell Biology, Harvard Medical School, Boston, MA 02115; ^5^ Science Park-Research Division, the University of Texas M. D. Anderson Cancer Center, Smithville, TX 78957

**Keywords:** longevity, FOXO transcription factors, methyltransferase, Set9, aging

## Abstract

The FoxO family of transcription factors plays an important role in longevity and tumor suppression by regulating the expression of a wide range of target genes. FoxO3 has recently been found to be associated with extreme longevity in humans and to regulate the homeostasis of adult stem cell pools in mammals, which may contribute to longevity. The activity of FoxO3 is controlled by a variety of post-translational modifications that have been proposed to form a ‘code’ affecting FoxO3 subcellular localization, DNA binding ability, protein-protein interactions and protein stability. Lysine methylation is a crucial post-translational modification on histones that regulates chromatin accessibility and is a key part of the ‘histone code’. However, whether lysine methylation plays a role in modulating FoxO3 activity has never been examined. Here we show that the methyltransferase Set9 directly methylates FoxO3 in vitro and in cells. Using a combination of tandem mass spectrometry and methyl-specific antibodies, we find that Set9 methylates FoxO3 at a single residue, lysine 271, a site previously known to be deacetylated by Sirt1. Methylation of FoxO3 by Set9 decreases FoxO3 protein stability, while moderately increasing FoxO3 transcriptional activity. The modulation of FoxO3 stability and activity by methylation may be critical for fine-tuning cellular responses to stress stimuli, which may in turn affect FoxO3's ability to promote tumor suppression and longevity.

## INTRODUCTION

FoxO transcription factors mediate longevity downstream of the insulin pathway in worms and flies [1-5]. In mammals, the role of FoxO factors in longevity has not been investigated yet, but mice with mutations in the insulin or IGF-1 receptors are long-lived [6, 7]. In addition, recent genetic studies on long-lived humans have shown that single nucleotide polymorphisms (SNPs) in the FoxO3 gene, one of the four human FoxO family members, are closely linked to extreme longevity and the reduction in age-related diseases [8-12]. These studies are consistent with the notion that mammalian FoxO3 may also be involved in regulating longevity. Importantly, FoxO transcription factors also act as tumor suppressors in mammals [13]. Indeed, mice in which the FoxO1, FoxO3, and FoxO4 genes are conditionally deleted in the adults develop thymic lymphomas and hemangiomas [14]. In humans, inactivation of FoxO3 correlates with poor prognosis in estrogen-dependent breast cancer [15, 16], illustrating the conserved role of the FoxO family in tumor suppression.

In mammalian cells, FoxO factors act as potent transcriptional activators that upregulate the expression of programs of genes involved in stress resistance, cell cycle arrest, differentiation, apoptosis, autophagy, and metabolism [17-30]. Recent evidence indicates that FoxO factors, and in particular FoxO3, is important for the maintenance of adult neural and hematopoietic stem cells [31-35]. However, much remains unknown about how FoxO transcription factors integrate extracellular stimuli to regulate different programs of gene expression and cellular responses that ultimately lead to lifespan extension and tumor suppression.

FoxO transcription factors are regulated by a wide array of external stimuli, ranging from insulin and growth factors to oxidative and nutrient stresses [36], as well as a variety of chemical activators such as LY294002 [37] and hypomethylating agents such as azacitidine and decitabine [38]. The principal mode of regulation of FoxO factors is via post-translational modifications (PTMs). Phosphorylation by the insulin-activated protein kinases Akt and SGK at three conserved sites on FoxO factors leads to the sequestration of FoxO in the cytoplasm, thereby preventing FoxO factors from transactivating their target genes [18, 39-42]. Phosphorylation of FoxO factors in response to insulin pathway stimulation and to cytokines also promotes the degradation of FoxO factors by the ubiquitin-proteasome pathway [15, 43-46].

FoxO factors are post-translationally modified at a number of lysine residues by ubiquitination and acetylation. For example, FoxO1 is polyubiquitinated by the SCFskp2 complex [46], and FoxO3 is polyubiquitinated by MDM2 and Skp-2 [47, 48], prior to degradation. FoxO factors can also be mono-ubiquitinated, which promotes FoxO nuclear translocation and regulates FoxO transcriptional activity [49, 50]. Acetylation/deacetylation of FoxO factors at a number of lysine residues also regulates FoxO subcellular localization, DNA binding capacity and transcriptional activity towards specific target genes [51-61]. The large number and diversity of PTMs on FoxO factors have lead us to hypothesize that these PTMs form a ‘FoxO code’ that could regulate several aspects of FoxO activity [62].

Methylation of lysine residues is a critical PTM for the regulation of histone proteins in chromatin [63]. There are about 50 lysine methyltransferases in mammals [64, 65]. The Su(var), Enhancer of zeste, and Trithorax [28] -domain containing lysine methyltransferases are conserved throughout species and catalyze a variety of histone methylations that are critical in regulating chromatin structure. Interestingly, some lysine methyltransferases can also methylate non-histone proteins. For example, Set9 (also known as Set7/9 and KMT7) was first identified as a mono-methyltransferase for lysine 4 in Histone H3 (H3K4) [66], but Set9 also methylates several non-histone proteins, including transcription factors (RelA (p65), p53, ERa), enzymes (DNMT1, PCAF), and transcription-initiation proteins (TAF10) [67-73]. Set9 methylation has been shown to affect the protein stability of Rel, p53, ERa and DNMT1 [67-69, 73, 74], and to alter the recruitment of transcription factors and transcriptional machinery such as RelA and TAF10 to different promoters [71, 72]. These observations suggest that Set9 has a complex role beyond chromatin regulation.

The importance of Set9 in the organism is underscored by the observation that half of the Set9 knockout mice die prior to birth [75]. Mouse embryonic fibroblasts (MEFs) from Set9 heterozygous and null mice are more susceptible to transformation than wildtype MEFs, suggesting that Set9, like FoxO3, acts as a tumor suppressor [75]. This observation, coupled with the role of Set9 in a variety of cellular processes also regulated by FoxO3, including cell cycle arrest [69, 75] and apoptosis [69], and the activation of Set9 by stress stimuli [69, 74], raised the possibility of a connection between Set9 and FoxO3.

Here we find that the lysine methyltransferase Set9 methylates FoxO3 directly *in vitro* and in cells. We identify a single lysine residue methylated by Set9 on FoxO3. This residue is important in modulating the transcriptional activity of FoxO3 and its stability. In addition to uncovering a novel non-histone substrate for Set9, our study identifies lysine methylation as an additional post-translational modification of FoxO3 that is likely part of the ‘code’ that modulates FoxO3's activity in response to environmental stimuli. Our findings further our understanding of the regulation of a critical transcription factor involved in longevity and cancer, and expand our knowledge of the role of Set9 in cells.

## RESULTS

### FoxO3 is Methylated by Set9 *in Vitro*

**Figure 1:**
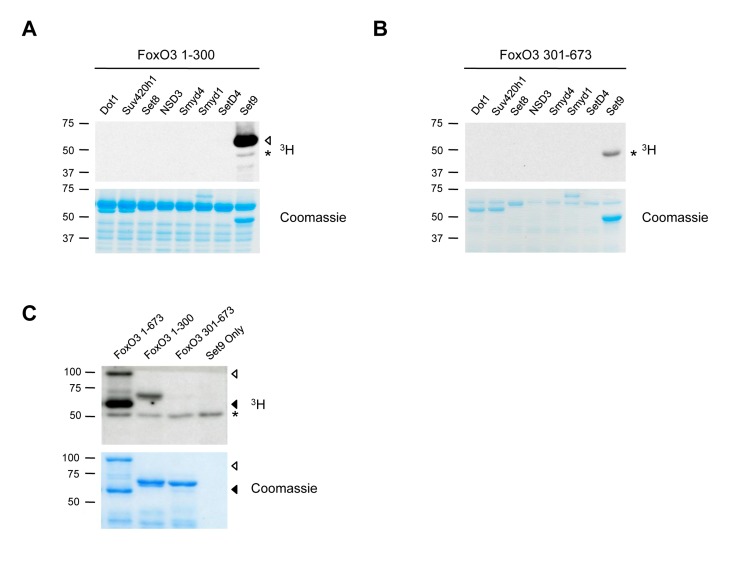
FoxO3 is methylated by Set9 in vitro (**A**) *In vitro* methylation of the N-terminal portion of FoxO3 (amino acids 1-300) by 8 different methyltransferases. ◁: FoxO3 methylated by Set9, *: Set9 auto-methylation. (**B**) In vitro methylation of the C-terminal portion of FoxO3 (amino acids 301-673) by 8 different methyltransferases. *: Set9 auto-methylation. (**C**) Methylation of the full-length FoxO3 protein by Set9. ◁: full-length FoxO3, ◀: FoxO3 degradation product, *: Set9 auto-methylation.

Among the four FoxO family members, FoxO3 is the isoform that has been associated with human longevity [8-11], breast cancer suppression in humans [15, 16], and in the maintenance of adult hematopoietic and neural stem cells [31, 34], a potentially important process in maintaining health late in life. Thus, we focused on FoxO3, and screened for lysine methyltransferases that could methylate the N-terminal or C-terminal portions of FoxO3 in an *in vitro* methylation assay (Fig. 1A, B). We found that among eight methyltransferases, only Set9, a member of the Set domain-containing lysine methyltransferase family, methylated the N-terminal domain of FoxO3 (Fig. 1A). We confirmed that full-length FoxO3 was methylated by Set9, and that only the N-terminal portion (1-300) of FoxO3 was methylated by Set9 (Fig. 1A-C). These results indicate that FoxO3 is a substrate of Set9 *in vitro* and that the site of methylation is located between amino acids 1-300 of FoxO3.

### FoxO3 is Methylated by Set9 at Lysine 271 *in Vitro*

**Figure 2:**
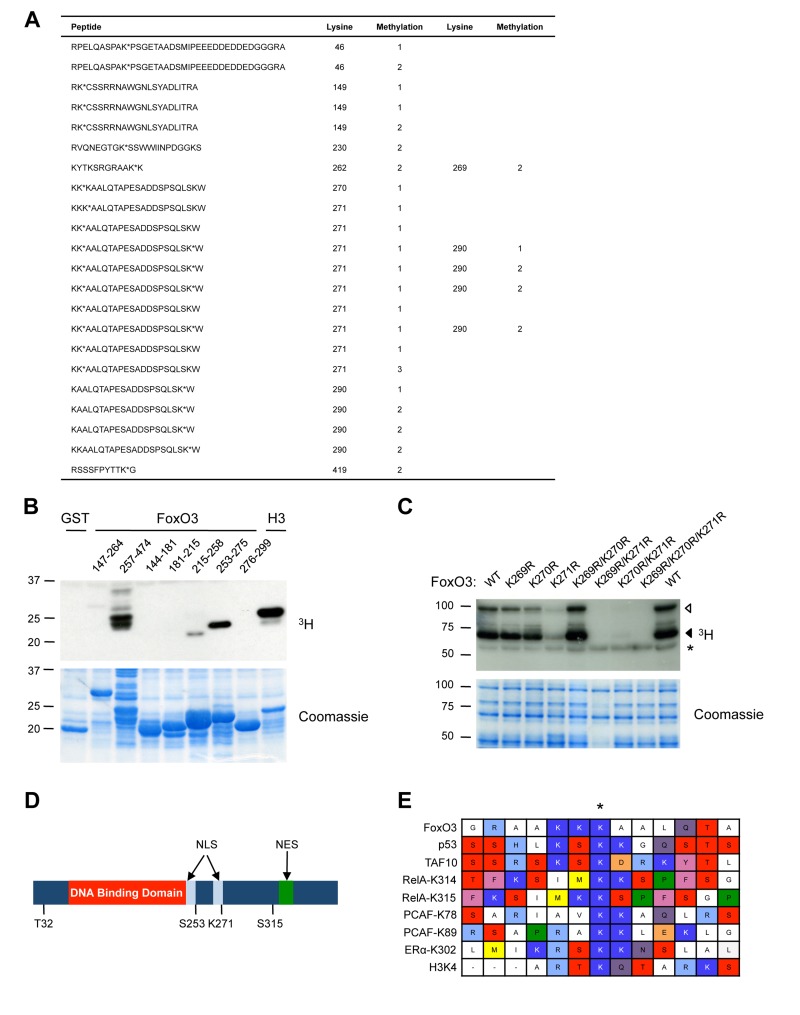
Set9 mono-methylates FoxO3 at K271 in vitro (**A**) Tandem mass spectrometry on *in vitro* methylated full-length FoxO3. Peptides containing methylated lysines are shown and the number of the methylated lysine in the human FoxO3 amino acid sequence is indicated. Methylated lysines are followed by an *. The type of methylation (mono-, di-, or tri-) is indicated by 1, 2, or 3, respectively. (**B**) Deletion analysis to map the region of FoxO3 containing the methylation site. *In vitro* methylation of overlapping fragments spanning the N-terminal domain of FoxO3 by Set9. (**C**) Methylation of FoxO3 WT or mutants of specific lysine residues. Each mutant was made in the context of the GST-FoxO3 protein (amino acid 1-525). ◁: FoxO3, ◀: FoxO3 degradation product, *: Set9 auto-methylation. (**D**) Location of K271 compared to other domains and PTMs of FoxO3. Listed are Akt phosphorylation sites (T32, S253, and S315), DNA binding domain, and NLS and NES (nuclear export sequence). K271 is the final amino acid in the second part of the bipartite FoxO3 NLS. (**E**) Alignment of the region surrounding the residues methylated by Set9 in a series of known Set9 substrates.

To identify the site(s) of methylation of FoxO3 by Set9, we analyzed the peptides generated by enzymatic digest of full-length FoxO3 that had been methylated by Set9 *in vitro* using tandem mass spectrometry (Fig. 2A). This tandem mass spectrometry analysis revealed that 9 lysines of FoxO3 were methylated *in vitro* by Set9: K46, K149, K230, K262, K269, K270, K271, K290, K419. With the exception of K419, all the sites of methylation identified by mass spectrometry were located between amino acids 1-300 of FoxO3, consistent with our observation that this portion of FoxO3 was the one methylated by Set9 (see Fig. 1). Based on the number of peptides identified, mono-methylation of K271 was the most prominent post-translational modification of FoxO3 by Set9 (Fig. 2A, peptides in bold). K290 was also found on multiple peptides to be mono- or di-methylated. However, because Set9 has been reported to be capable of only mono-methylating its substrates due to the structure of the active site [76], it is possible that the di-methylation is an artefact of tandem mass spectrometry.

To identify in an independent manner the main site(s) of FoxO3 methylated by Set9, we used a deletion approach. We found that the regions between amino acids 257-474 and 253-275 were heavily methylated by Set9, whereas the region between 215-258 only displayed trace levels of methylation by Set9 (Fig. 2B). There are five lysine residues in the portion of FoxO3 comprised between amino acids 257-275, four of which that have also been identified by tandem mass spectrometry as methylated by Set9 (K262, K269, K270, K271). In contrast, the regions between amino acids 144-215, and 276-299 were not methylated at all by Set9 (Fig. 2B), suggesting that K149 and K290 are not major methylation sites in FoxO3, even though there were identified by mass spectrometry.

To identify the main residue of FoxO3 methylated by Set9, we generated point mutants of FoxO3 for which K269, K270, or K271 were replaced by an arginine, either individually or concomitantly. We compared the *in vitro* methylation by Set9 of wildtype (WT) FoxO3 with that of each FoxO3 mutant. The FoxO3 mutant in which lysine 271 was replaced by an arginine (K271R) was the only mutant that showed a significant decrease in methylation by Set9 (Fig. 2C). In addition, the double mutants that contained K271R (K269R/K271R and K270R/K271R) were no longer methylated by Set9, whereas the double K269R/K270R mutant showed levels of methylation by Set9 that were similar to that of WT FoxO3 (Fig. 2C). K262R and K290R mutants did not display lower levels of methylation as compared to wild type FoxO3 (data not shown). Taken together, these results indicate that lysine 271, a residue located in the second half of the bipartite nuclear localization sequence (NLS) of FoxO3 [77], is the primary methylation site on FoxO3 by Set9 *in vitro* (Fig. 2D).

To determine if the amino acids surrounding K271 formed a potential consensus sequence for Set9 methylation, we aligned a 13 amino acid region surrounding this site in FoxO3 with regions of similar length surrounding the Set9-methylated lysine residues in previously identified substrates (Fig. 2E). This alignment did not reveal a clear consensus sequence for Set9 methylated sites, but it suggested that Set9 requires positively charged residues (K or R) in the vicinity of the methylated lysine (Fig. 2E).

### FoxO3 is the FoxO Family Member Most Strongly Methylated by Set9

**Figure 3:**
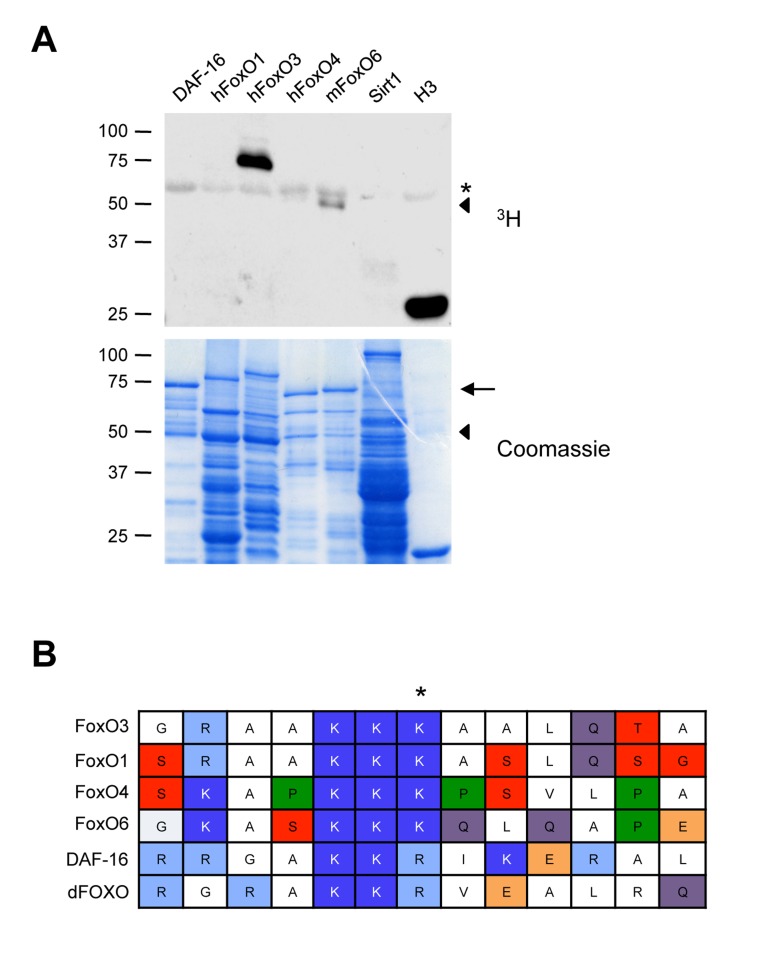
Set9 preferentially methylates FoxO3 among FoxO family members (**A**) *In vitro* methylation of different mammalian FoxO family members or FoxO orthologs. The FoxO1, FoxO3 and FoxO4 constructs were generated from the human sequence, whereas the FoxO6 construct was generated from the mouse sequence. ◀: FoxO6 degradation product, ←: full-length FoxO6, *: Set9 auto-methylation. (**B**) Alignment of the region of FoxO3 surrounding K271 to that of other mammalian FoxO family members and FoxO orthologs.

We next asked if Set9 also methylated other mammalian FoxO family members (human FoxO1, human FoxO4, and mouse FoxO6), as well as FoxO orthologs in other species (DAF-16, the worm FoxO). While we do see methylation of other mammalian FoxO family members (most noteably a fragment of mouse FoxO6) as well as worm ortholog DAF-16, surprisingly, FoxO3 was the only protein in the FoxO family that was robustly methylated by Set9 under the conditions used (Fig. 3A).

To determine if there was a particularity in FoxO3 sequence underlying the specificity of Set9 towards FoxO3, we aligned the region surrounding FoxO3 K271 with that of other mammalian and non-mammalian FoxO family members. This analysis revealed that the lysine residues corresponding to K271 are conserved in all mammalian FoxO family members (Fig. 3B), suggesting that there are other parameters, such as secondary structure, that may influence the relative specificity of Set9 for FoxO3.

### FoxO3 is Methylated by Set9 at K271 in Human Cells

**Figure 4:**
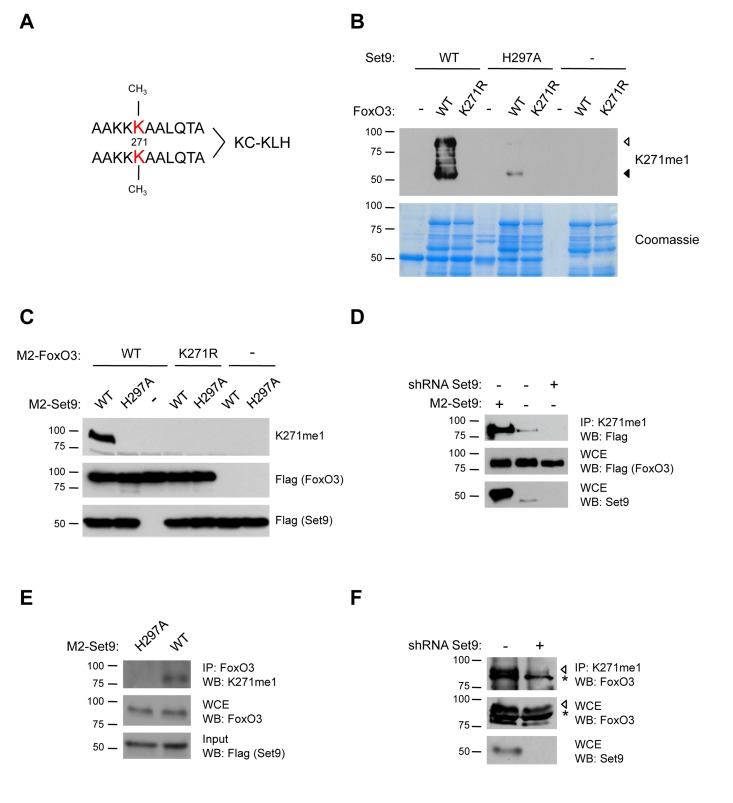
FoxO3 is methylated at K271 in cells (**A**) Scheme for generation of K271me1 antibody. A branched mono-methylated 11 amino acid peptide surrounding FoxO3 K271 was used as the epitope. (**B**) The K271me1 antibody is specific to FoxO3 that has been methylated at K271 in *in vitro* methylation assays. *In vitro* methylation assays were conducted with cold SAM and analyzed by western-blot with the K271me1 antibody [33]. Coomassie staining was used to show equal levels of FoxO3 and Set9 were used in each condition (bottom). ◁: FoxO3, ◀: FoxO3 degradation product. (**C**) The K271me1 antibody is specific to FoxO3 K271 methylation in cells. Flag-FoxO3 and Flag-Set9 (WT or H297A methyltransferase-deficient mutant) were co-expressed in 293T cells, and FoxO3 methylation was analyzed by western-blot with the K271me1 antibody. (**D**) Endogenous Set9 methylates ectopically expressed FoxO3. The methylated form of FoxO3 was immunoprecipitated from a 293T cell line with a stable knock-down of Set9 using the K271me1 antibody and the IP eluates were analyzed by western-blot with the Flag antibody. (**E**) Endogenous FoxO3 is methylated by overexpressed Set9. 293T cells were transfected with Flag-Set9, and FoxO3 was immunoprecipitated with an antibody to total FoxO3. The IP eluates were analyzed by western-blot with the K271me1 antibody. (**F**) Endogenous FoxO3 is methylated by endogenous Set9. Methylated FoxO3 was immunoprecipitated from the stable Set9 shRNA cell line used in (**D**) using the K271me1 antibody. The IP eluate was analyzed by western-blot with an antibody to total FoxO3. ◁: FoxO3, *: non-specific band.

To investigate whether FoxO3 is methylated by Set9 in mammalian cells, we generated an antibody specifically directed to the mono-methylated lysine 271 of FoxO3 (K271me1). As an epitope, we used a branched peptide that contains two mono-methylated epitopes per molecule (Fig. 4A). To test the specificity of this antibody to methylated FoxO3, we performed an *in vitro* methylation assay with wild type (WT) FoxO3 or the FoxO3 K271R mutant as substrates, and either WT Set9 or a methyltransferase-deficient version of Set9 (H297A) as the enzyme [66]. The K271me1 antibody recognized only WT FoxO3 that was incubated with WT Set9, and did not recognize FoxO3 K271R mutant (Fig. 4B). The K271me1 antibody did not recognize FoxO3 in the presence of the methyltransferase-deficient mutant of Set9, confirming the specificity of this antibody (Fig. 4B). There was a minor signal from a degradation fragment of FoxO3 in the presence of the Set9 methyltransferase-deficient mutant, which might be due to the fact that this mutant is not completely deficient for methyltransferase activity *in vitro*. Together, these results indicate that the antibody is specific for methylation of K271 on FoxO3 *in vitro*.

To test whether FoxO3 was methylated by Set9 in mammalian cells, we co-transfected human 293T cells with constructs encoding Flag-tagged versions of FoxO3 and Set9, and examined FoxO3 methylation by western-blot with the K271me1 antibody. We found that the expression of Flag-Set9 led to an increased methylation of WT FoxO3 (Fig. 4C). In contrast, when the methyltransferase-deficient Set9 mutant (H297A) was co-expressed with FoxO3, FoxO3 was no longer recognized by the K271me1 antibody (Fig. 4C). Furthermore, a mutant of FoxO3 containing a point mutation for the methylation site identified *in vitro* (K271R) was not recognized by the antibody to K271me1, even when co-expressed with WT Set9 (Fig. 4C). Taken together, these results confirm that the antibody to K271me1 we generated is specific, and indicate that overexpressed Set9 can methylate FoxO3 at lysine 271 in mammalian cells.

To examine if endogenous Set9 is necessary for FoxO3 methylation in 293T cells, we generated a stable cell line in which an shRNA against Set9 was introduced by viral transduction. The efficiency of Set9 knock-down in this cell line was confirmed by western-blot with an antibody to endogenous Set9 (Fig. 4D). To assess the methylation level of FoxO3 in the Set9 knock-down stable cell line, we immunoprecipitated Flag-FoxO3 in this cell line or in control cells and performed western-blot with the K271me1 antibody. We found that this antibody recognized a band at the molecular weight of FoxO3 in cells overexpressing FoxO3 alone in the control cell line and that this signal was reduced in the stable cell line with a Set9 shRNA knock-down (Fig. 4D). These results indicate that endogenous Set9 is necessary for the methylation of overexpressed Flag-FoxO3.

Conversely, to determine if endogenous FoxO3 is methylated by Set9 in mammalian cells, we overexpressed WT Set9 or the methyltransferase-deficient mutant of Set9 (H297A) in 293T cells and immunoprecipitated endogenous FoxO3 from these cells with an antibody to FoxO3 (Fig. 4E). We then assessed if endogenous FoxO3 was methylated using the K271me1 antibody in western-blot experiments. This antibody detected a band at the appropriate molecular weight for FoxO3 when FoxO3 was immunoprecipitated from cells expressing WT Set9, but not from cells expressing the inactive H297A mutant of Set9 (Fig. 4E). These data indicate that endogenous FoxO3 is methylated by overexpressed Set9.

Finally, to test if endogenous FoxO3 is methylated in cells by endogenous Set9, we used the stable cell line with Set9 knock-down. Protein lysates from these cells and control cells were subjected to immunoprecipitation with the K271me1 FoxO3 antibody and analyzed by western-blot using the antibody to total FoxO3 (Fig. 4F). These experiments revealed a band that corresponds to FoxO3 in the control cell line and the intensity of this band was reduced in the cell line with knocked down Set9 (Fig. 4F), indicating that endogenous FoxO3 is methylated by endogenous Set9 in cells.

Taken together, these experiments indicate that FoxO3 is methylated by Set9 in the human 293T cell line, and that Set9 is necessary and sufficient for lysine 271 mono-methylation on FoxO3.

### The Methylated Form of FoxO3 Has Similar Localization to Total FoxO3

**Figure 5:**
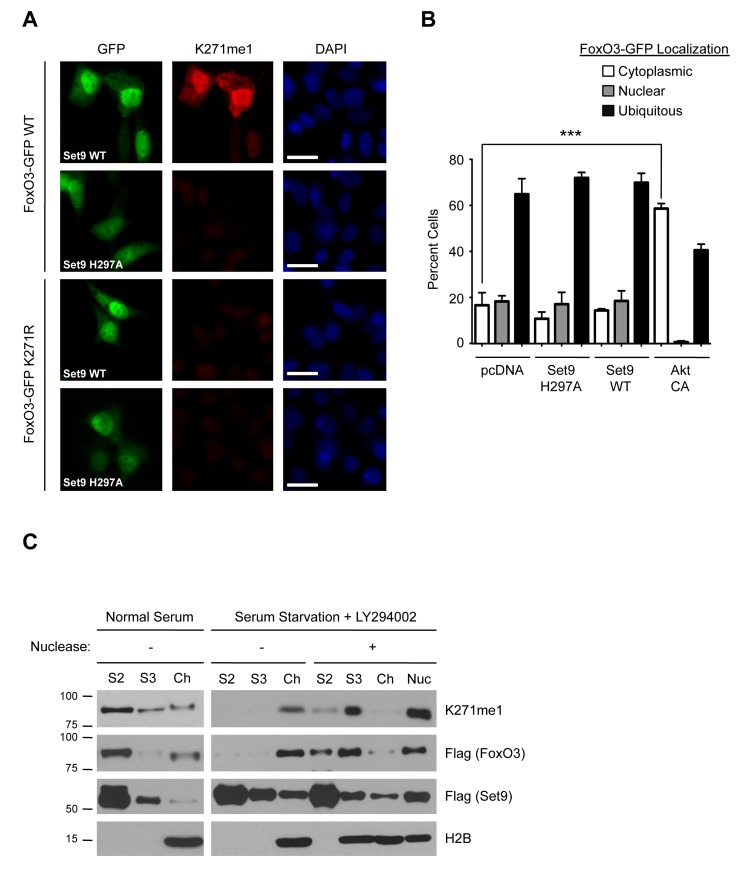
The methylated form of FoxO3 has similar localization properties to FoxO3 (**A**) Immunofluorescence in NIH 3T3 cells expressing FoxO3-GFP (green), using the K271me1 antibody (red). Nuclei were stained with DAPI (blue). White bar: 10 mm. (**B**) Quantification of FoxO3-GFP localization in U87 cells. Cells were co-transfected with FoxO3-GFP and empty vector (pcDNA), Set9 (WT or H297A), or a constitutively active version of Akt as a positive control (Akt CA). Localization was scored as ‘nuclear’, ‘cytoplasmic’ or ‘ubiquitous’. The data represent the mean and SEM from 3 independent experiments. In each experiment, at least 200 cells per condition were scored. *** p<0.001, one way ANOVA, Bonferroni post-test (**C**) Chromatin fractionation of 293T cells co-transfected with Flag-FoxO3 and Flag-Set9. Cells were grown in media with 10% FBS (Normal Serum) or in media without serum in the presence of 20 μM LY294002 (Serum Starvation+LY294002) and then fractionated. A subset of the fractions was treated with micrococcal nuclease to digest the chromatin. Presence of chromatin-bound proteins in fractions was assessed by western-blot with antibodies to the core nucleosome histone H2B (H2B). S2: detergent lysis, S3: hypotonic lysis fraction, Ch: chromatin fraction, Nuc: supernatant from nuclease digest of chromatin.

To examine the localization of methylated FoxO3 in cells, we conducted immunofluorescence experiments on NIH 3T3 cells expressing FoxO3-GFP using the K271me1 antibody. When Set9 was co-transfected with FoxO3-GFP, the K271me1 antibody detected a signal in FoxO3-GFP expressing cells (Fig. 5A). The signal from the K271me1 was significantly reduced when either the methyltransferase-deficient mutant of Set9 (H297A) was expressed or when cells expressed the K271R mutant of FoxO3, indicating that the immuno-fluorescence signal was specific for Set9 methylation of K271 (Fig. 5A). The localization of the methylated form of FoxO3 overlapped with that of total FoxO3, suggesting that methylation does not influence FoxO3 subcellular localization.

To further test whether methylation by Set9 affects FoxO3 localization, we transfected a human glioblastoma cell line (U87) with FoxO3-GFP and either Set9 WT or the methyltransferase-deficient mutant of Set9 (H297A). We chose the U87 cell line because these cells have high transfection efficiency and a relatively flat morphology, which is helpful to assess FoxO3 subcellular localization. FoxO3-GFP localization was scored ‘nuclear’ if the nucleus was clearly identifiable in the GFP channel, ‘cytoplasmic’ if the exclusion from the nucleus was visible in the GFP channel, and ‘ubiquitous’ if neither of these conditions was met. As expected, a constitutively active version of the protein kinase Akt significantly increased the cyto-plasmic localization of FoxO3-GFP (Fig. 5B). In contrast, WT Set9 expression did not significantly affect the localization of FoxO3-GFP, as compared to expression of the methyltransferase-defective H297A Set9 mutant or the empty vector (Fig. 5B). Thus, methylation by Set9 does not appear to affect FoxO3 subcellular localization in U87 cells.

### The Methylated Form of FoxO3 Associates with Chromatin Upon FoxO3 Activation

We next sought to determine if methyl-FoxO3 is associated with the chromatin. We co-expressed Flag-FoxO3 with Flag-Set9 in 293T cells under basal conditions or under conditions that are known to activate FoxO3 (serum starvation in the presence of the PI3K inhibitor LY294002 [18]), and we performed cellular fractionation experiments to separate the non-soluble chromatin fraction (Ch) from the soluble, non-chromatin fractions (S2 and S3). Under basal conditions, we observed that total FoxO3 is primarily present in the non-chromatin fraction (S2), with very little FoxO3 in the chromatin fraction (Fig. 5C). Western-blotting with the K271me1 antibody revealed that the distribution of the methylated form of FoxO3 was similar to that of total FoxO3, with most of the signal present in the S2 fraction. Upon conditions that activate FoxO3, we found that total FoxO3 relocalized to the chromatin fraction (Fig. 5C). The methylated form of FoxO3 also relocalized to the chromatin fraction. Nuclease treatment of the chromatin fraction resulted in a shift in the distribution of FoxO3 from the chromatin fraction to the S3 fraction, indicating that FoxO3, and methyl-FoxO3, were bound to the chromatin and not to membrane proteins or other cell components in the chromatin pellet. These results indicate that FoxO3 relocalizes to the chromatin fraction upon activation and that the methylated form of FoxO3 is also found at the chromatin.

### Methylation of FoxO3 Does Not Cause Increased Association with Methyl-Lysine Binding Proteins

**Figure 6:**
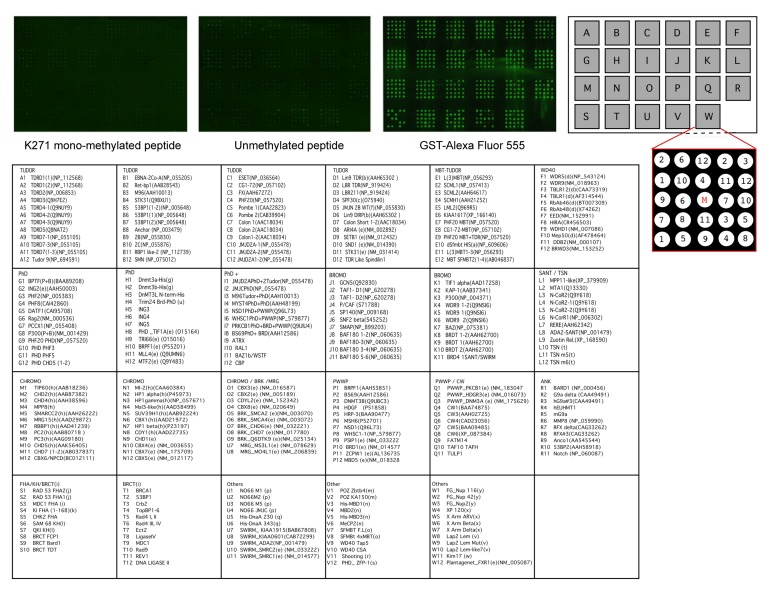
The methylated K271 peptide does not bind to kown methyl-lysine binding domains Twenty-one amino acid peptides surrounding K271, either mono-methylated (left) or unmethylated (right) were incubated with potential methyl-lysine and acetyl-lysine binding domains on a protein array. GST-Alexa Fluor 555 is used as a positive control. A detailed description of the binding domains present on the protein array is provided in the lower panels.

Because methyl-FoxO3 is localized to the chromatin under after serum starvation and LY294002 treatment, we asked if binding proteins that are known to bind to methylated histones could interact with the methylated fraction of FoxO3 (Fig. 6). We used a protein array that contained 265 domains found in chromatin associated proteins [78], many of which are known methyl-lysine binding domains (including TUDOR domains, PhD domains, and chromo-domains) or acetyl-lysine binding domains (including bromo-domains, for a complete list see Fig. 6, lower panels). We probed this protein array with a biotinylated 21 amino acid peptide surrounding K271 of FoxO3 either non-methylated (unmethylated peptide) or mono-methylated at K271 (K271 mono-methylated peptide). Peptide binding to the methyl-lysine binding domains on the array was assessed by immunofluorescence with antibodies to the biotin moiety. We did not observe binding of the K271 mono-methylated peptide for any of the domains present on the protein array (Fig. 6). These data suggest that mono-methylation of FoxO3 does not induce the binding to any of these chromatin-binding domains, although we cannot exclude that in the context of the whole protein, methylated FoxO3 may bind to chromatin-binding proteins that ‘read’ histone marks.

### Methylation by Set9 Decreases the Stability of the FoxO3 protein

**Figure 7:**
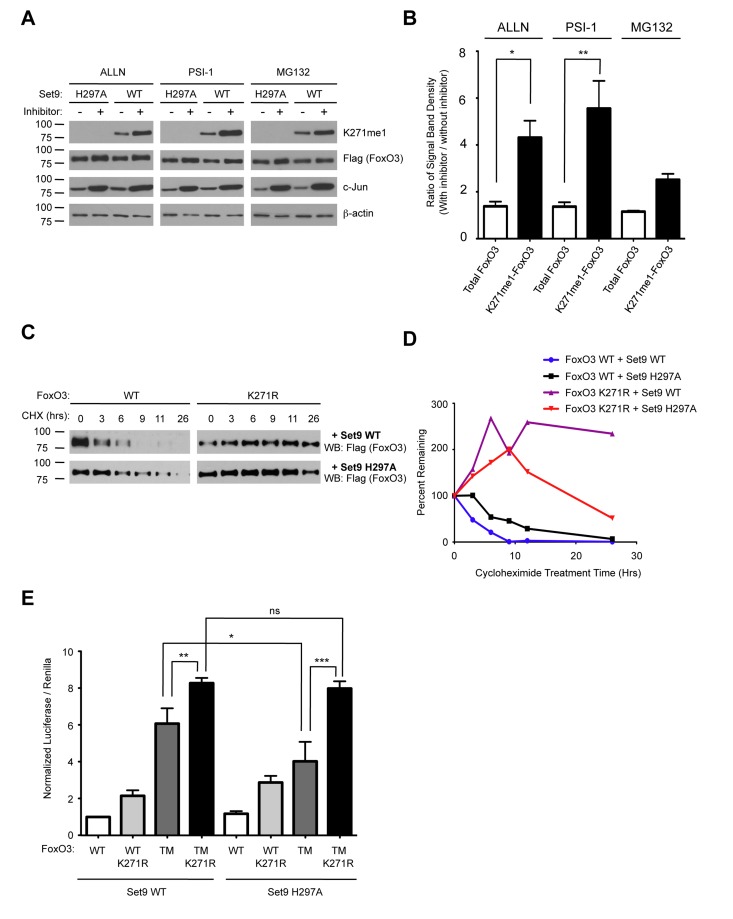
Methylation of FoxO3 at lysine 271 promotes FoxO3 degradation, while increasing FoxO3 transcriptional activity (**A**) Methylated FoxO3 is less stable than the unmethylated form. 293T cells were co-transfected with Flag-FoxO3 and Flag-Set9 (WT or H297A). The cells were then treated with MG132 (20 μM), ALLN (40 μM) or PSI1 (40μM) for 16 hours prior to lysis. Extracts were analyzed by western-blot with antibodies to K271me1, Flag, c-Jun, and b-actin. (**B**) Western-blots from (**A**) were analyzed by densitometry and the ratio of intensity in the presence or absence of proteasome inhibitor was compared after normalization to b-actin. * p< 0.05, **p<0.01, One way ANOVA, Bonferroni post test. (**C**) K271R is more stable than wild type FoxO3. 293T cells were transfected with Flag-FoxO3 (WT or K271R) together with Flag-Set9 (WT or H297A). Cells were treated for the indicated length of time with 20 μM cycloheximide (CHX) and FoxO3 expression was tested by western-blot with antibodies to Flag. (**D**) Western-blots from (**C**) were analyzed by densitometry at each time point of cycloheximide treatment. Results were normalized to the untreated well for each condition. (**E**) Set9 increases the transcriptional activity of the constitutively nuclear form of FoxO3. Flag-Set9 (WT or H297A) was co-expressed with Flag-FoxO3 (WT, K271R, TM [T32A/S253A/S315A], or TM K271R [T32A/S253A/K271R/S315A]) in 293T cells together with a 6xDBE-luciferase reporter construct, and luciferase activity was measured. The data represents the mean and SEM from 5 independent experiments, conducted in triplicate. All samples were normalized to the Flag-FoxO3 WT + Flag-Set9 WT condition. * p< 0.05, ** p< 0.01, *** p< 0.001, ns: p> 0.05, One way ANOVA, Bonferroni post test.

Methylation by Set9 has been shown to affect the protein stability of RelA, Era, DNMT1 and p53 [67-69, 73, 74]. To test the possibility that Set9 methylation affected FoxO3 protein stability, we co-transfected wild type FoxO3 together with either WT Set9 or the methyltransferase-deficient mutant of Set9 (H297A) in 293T cells. We then treated the cells with a series of different proteasome inhibitors: ALLN, Proteasome Inhibitor 1 (PSI-1), and MG132. The levels of c-Jun, a protein known to be degraded via a proteasome dependent pathway was increased upon treatment with proteasome inhibitors, indicating that these inhibitors were indeed effective at blocking the proteasome (Fig. 7A). We found that treatment of cells with proteasome inhibitors caused both total FoxO3 and methyl-FoxO3 levels to increase. Interestingly, densitometry measurements of the relative amount of FoxO3 levels normalized to β-actin levels showed an increase in the methyl-FoxO3 levels compared to total levels of FoxO3 when cells were treated with proteasome inhibitors (Fig. 7A-B). Although the effects of MG132 on FoxO3 were not statistically significant, there was a trend towards increased relative levels of methylated FoxO3 in the presence of MG132 (Fig. 7B). Together, these results suggest that the methylated form of FoxO3 is less stable than the non-methylated version of FoxO3.

### FoxO3 K271R Mutant is More Stable than Wild Type FoxO3

To further explore whether methylation at lysine 271 might control the stability of FoxO3, we transfected 293T cells with WT FoxO3 or the K271R mutant form of FoxO3 together with WT or H297A Set9, and treated cells with cycloheximide to block de novo translation. Interestingly, we found that FoxO3 K271R was more stable than WT FoxO3 (Fig. 7C-D). We also found that WT FoxO3 was slightly less stable when co-expressed with WT Set9 as compared to H297A Set9 (Fig. 7C-D). These results suggest that methylation of lysine 271 is important for regulating the stability of FoxO3 in cells.

### Set9 Leads to a Modest Increase in Nuclear FoxO3 Transcriptional Activity

To test if Set9 affected FoxO3 transcriptional activity, we co-transfected 293T cells with Set9 (WT or methyltransferase-deficient mutant [H297A]), together with FoxO3 (WT, K271R, a constitutively nuclear mutant in which all three Akt phosphorylation sites have been converted to alanines [TM], and the combined mutant TM K271R), and with a luciferase reporter construct that contained 6 FoxO DNA binding elements (6xDBE) (Fig. 7E). Co-expressing Set9 WT together with the constitutively nuclear form of FoxO3 (FoxO3 TM) led to a modest, but significant increase in the luciferase reporter activity compared to co-expression of the methyltransferase-deficient form of Set9 and FoxO3 TM (Fig. 7E). This result suggests that Set9 increases FoxO3 transcriptional activity. The TM K271R mutant of FoxO3 had an increased luciferase activity compared to TM FoxO3, probably because mutating K271 increased stability of the TM mutant (Fig. 7E). However, Set9 did not further increase luciferase levels when the TM K271R mutant of FoxO3 was coexpressed (Fig. 7E), suggesting that methylation at K271 is necessary for the activation of FoxO3 mediated transcriptional activation.

Taken together, these studies indicate that FoxO3 is methylated at lysine 271 by Set9, and that this modification does not affect FoxO3 subcellular localization, but decreases FoxO3 protein stability while eliciting a modest increase in FoxO3 transcriptional activity.

## DISCUSSION

Lysine methylation of non-histone proteins has been recently found to be critical for a number of cellular responses, including the response to stress stimuli [68, 69, 72, 74]. In several cases, methylation affects the status of other post-translational modifications (PTMs) present on the target protein [72]. Lysine 271, the Set9 methylation site on FoxO3 has been previously shown to be acetylated [54]. Thus, methylation at FoxO3 lysine 271 could block acetylation at this site. It is possible that the competition for modifying lysine 271 could partly control FoxO3-dependent responses to stress stimuli. Like other PTMs, FoxO3 K271 methylation might be regulated by external stimuli. Indeed, we have found that oxidative stress can change the level of FoxO3 K271 methylation under certain conditions (data not shown). It is possible that the activity of protein deacetylases, such as Sirt1, which can deacetylate K271 (A.B., unpublished data), could facilitate methylation at this residue and therefore act in partnership with Set9 to regulate the presence of the methylated form of FoxO3 at the chromatin and increase FoxO3 transcriptional activity.

The conservation of lysine 271 and the residues surrounding this lysine in all FoxO family members, and yet the absence of significant methylation of FoxO1, FoxO4, and FoxO6 by Set9 indicates that the mechanism of target identification by Set9 could be more complex than a simple consensus sequence. Comparison of the known Set9 targets does not display a clear consensus for residues surrounding the lysine residue that is methylated. All of the targets have at least one positively charged residue N- or C-terminal to the site of methylation, so the active site of Set9 could require a local positive charge interaction for methylation or the positively charged residue could be important for hydrogen bonding with a residue in Set9. This was shown to be the case for p53 [69]. In addition, all of the substrates have a negatively charged residue at position +4 or +5 compared to the methylation site, and this negative charge could be important in positioning the substrate for methylation. However, given the lack of a strong consensus sequence, it is probable that the substrates of Set9 need to have a secondary structure that is not immediately identifiable from the amino acid sequence. The Set9 crystal structure with a p53 peptide was found to be similar to that of the Set9 crystal structure with a mono-methyl H3 peptide [69, 76]. The necessity of the lysine side chain of the residue to be methylated to enter a narrow channel in order to access the methyl-donor cofactor illustrates how important secondary structure of the substrate is for Set9 activity. It is possible that the substrate must have a flexible secondary structure, much like a histone tail, in order to allow the lysine side chain access to this channel within Set9.

The regulation of FoxO3 activity by methylation could be pivotal to precisely modulate FoxO3's activity in response to stress. The increase in FoxO3 activity combined with a decrease in stability could create a transient pulse of FoxO3 activity intended to upregulate genes to mount an immediate response to stress. An increase in activity and decrease in stability has also been seen as a consequence of deacetylation on the FoxO family [59], consistent with a possible coordinated action of deacetylation and methylation. Mutations that increase the transcriptional activity of FoxO proteins without decreasing FoxO stability often lead to apoptosis (as is the case with the TM mutation). Thus, a transient pulse of FoxO activity may preferentially lead to the upregulation of stress resistance genes, without resulting in the upregulation of apoptosis genes.

Lysine methylation of FoxO3 represents a previously unidentified type of post-translational modification that adds to the growing number of PTMs on this transcription factor. Together, FoxO3 PTMs may form a ‘code’ for fine-tuning the activity of this transcription factor in cells. Interestingly, there seem to be interaction between the different FoxO3 PTMs. For example, arginine methylation of FoxO1 by PRMT1 has recently been described to inhibit the phosphorylation, and subsequent degradation, of FoxO1 by Akt [79]. Much like lysine methylation, arginine methylation can have a potent impact on the transcriptional activity of non-histone substrates [80]. It will be interesting to determine if lysine and arginine methylation both occur on the FoxO3 molecule and how they might interact with one another. The interaction between the different PTMs of FoxO3 at the same residue (e.g. acetylation/methylation/ubiquitination of a given lysine residue) or at different residues (e.g. lysine methylation versus arginine methylation) may alter several properties of the FoxO3 protein, including its stability, subcellular localization, recruitment to a subset of target genes, and interaction with protein partners.

Different PTMs might also serve to modulate FoxO3 activity differentially in different tissues and cellular environments. For example, a very recent study showed that Set9 also methylates FoxO3 at lysine 270, and that K270 methylation diminishes FoxO3's pro-apoptotic activity in neuronal cells by preventing FoxO3 binding to the promoter of the pro-apoptotic gene Bim [81]. This is in contrast with our study, since we find that methylation of FoxO3 at lysine 271 induces a modest increase in FoxO3 transcriptional activity - as measured by reporter assay - in 293T cells. It will be interesting to compare how methylation at lysine 270 and 271 modulate FoxO3 activity, and whether their effects on FoxO3 function is dependent on the cell type or the type of target genes.

Together with published data, our results indicate that FoxO3, a transcription factor critical for stress resistance and longevity in a wide range of species, has evolved to be a heavily post-translationally regulated molecule, with about thirty different post-translationally modified residues described so far. FoxO3 appears to be a ‘hub’ in a network of genes regulating cellular homeostasis and energy metabolism. Thus, the variety and complexity of FoxO3 PTMs may be critical for the interface between extrinsic stimuli and intrinsic networks that regulate plastic phenotypes, including stem cell homeostasis, that ultimately contribute to organismal longevity.

## EXPERIMENTAL PROCEDURES

### Antibodies

The FoxO3 antibody (‘NFL’) was described previously [18]. The Flag and Set9 antibodies were obtained from Sigma and Cell Signaling Technologies, respectively. The H2B antibody was obtained from Millipore. The c-Jun, and β-actin and GFP (3E6 monoclonal) antibodies were purchased from Santa Cruz, Novus Biologicals and Invitrogen, respectively. The mono-methyl-specific antibody was generated by Quality Controlled Biochemicals. A branched mono-methylated peptide encompassing human FoxO3 K271 (Fig. 4A) was synthesized by the Keck Biotechnology Resource Laboratory at Yale University. This peptide was coupled to Keyhole limpet hemocyanin and injected into rabbits for antibody production. The serum was purified on a streptavidin column bound to a biotin conjugated linear mono-methyl peptide synthesized by the Keck Biotechnology Resource Laboratory.

### Reagents

LY294002, ALLN and PSI-1 were all obtained from EMD Biosciences/Calbiochem. MG132 and Cycloheximide were purchased from Sigma.

### Constructs

The constructs encoding GST-FoxO1, GST-FoxO3, GST-FoxO4, and GST-FoxO6 in the pGex vector were described previously [18, 82]. The constructs encoding His-Set9 (WT and H297A) in the pET-28b vector, and Flag-Set9 (WT and H297A) in the pcDNA vector were described previously [66]. The Dot1, Suv420h1, and Set8 constructs were described previously [83]. The SetD4, Smyd4, Smyd1, and NSD3 were generated as fusion proteins between GST and the full-length human proteins and expressed in insect cells (X.S. and O.G.). The reporter construct 6xDBE-luciferase was described previously [84]. The constructs encoding WT FoxO3, the T32A/S253A/S315A (TM) mutant, and the K269R/K270R/K271R mutant in the pECE expression vector were described previously [18, 54, 77]. The construct encoding the constitutively active form of Akt (Akt CA) was described [18]. The FoxO3-GFP construct was described previously [15]. The FoxO3-GFP K271R mutant was generated using site-directed mutagenesis (Stratagene) using the forward primer GCGCAGCCAAGAAGAGGGCAGCCCTGCA GACAGC. The mutants of FoxO3 lysine residues were generated in the pECE vector using site directed mutagenesis (Stratagene) with the following primers: K269R:CGTGGCCGCGCAGCCAGGAAGAAGGCA GCCCTGC K270R:GGCCGCGCAGCCAAGAGGAAGGCAGCC CTGCAG K269R/K270R:GCCGTGGCCGCGCAGCCAGGAGG AAGGCAGCCCTGCAGACAGCC K269R/K271R:GCCGTGGCCGCGCAGCCAGGAAG AGGGCAGCCCTGCAGACAGCC K270R/K271R:CGTGGCCGCGCAGCCAAGAGGAG GGCAGCCCTGCAGACAGCC shRNA oligonucleotides directed at human Set9 were designed with pSico Oligomaker v1.5 (http://web.mit.edu/jacks-lab/protocols/pSico.html). shRNA Set9 F: TGAACTTTGTTCACGGAGAATTCAAGAGATTC TCCGTGAACAAAGTTCTTTTTTC shRNA Set9 R: TCGAGAAAAAAGAACTTTGTTCACGGAGAAT CTCTTGAATTCTCCGTGAACAAAGTTCA These oligonucleotides were annealed and cloned into pSicoR (PSR) between the HpaI and Xho1 sites according and annealed as described (http://web.mit.edu/jacks-lab/protocols/pSico.html).

### Protein purification

His-Set9 WT and H297A were purified using TALON Metal Affinity Resin according to the protocol provided by the manufacturer (BD Biosciences Clonetech). GST fusion proteins were purified on Glutathione agarose beads (Sigma), according to the manufacturer's protocol.

### Cell culture

HEK293T, U87, and NIH3T3 cells were maintained in Dulbecco Modified Eagle Medium (DMEM, Invitrogen) supplemented with 10% Fetal Bovine Serum (FBS, Invitrogen) and 1% Penicillin/Streptomycin/Glutamine (P/S/Q, Invitrogen). When indicated, cells were serum-starved by washing once with DMEM + 1% P/S/Q and then incubating them overnight in DMEM + 1% P/S/Q. Cells were then treated with the PI3K inhibitor LY294002 (EMD Biosciences, 20 μM) for 2 hours prior to lysing.

### Luciferase Assays

293T cells were seeded at the density of 1.25 × 105 cells per well in 24 well plates. In each well, 250 ng of the appropriate FoxO3 constructs and 50 ng of the Set9 constructs were co-transfected with 250 ng of the 6xDBE luciferase reporter constuct [84] and 50 ng of the Renilla control construct using TransIT-293 (Mirus, 2 μl per well). Twenty-four hours after transfection, cells were lysed in 100 μl of 1x passive lysis buffer (Promega). After centrifugation to remove cellular debris, the luminescence was measured in a Wallac Victor2 multilabel counter using the Dual-Luciferase reporter assay system (Promega), according to the manufacturer's protocol.

### *In Vitro* Methylation Assay

*In vitro* methylation assays were conducted in a total volume of 25 μl using 2 μg of substrate, 100 ng of Set9 WT or H297A, in the methylation buffer: 150 mM NaCl, 20mM Tris-HCl [pH 7.5], 1 mM EDTA, 0.02% Triton. Depending on the reaction, either 40 μM S-Adenosyl methionine (SAM, Sigma) or 300 nM S-[methyl-3H] Adenosyl-L-Methionine (Perkin Elmer, 0.55 μCi per reaction) was added. Reactions were carried out at 37°C for 3 hours to overnight. The reaction was resolved by SDS-PAGE (10%). After electrophoresis the gel was incubated with EN3HANCE™ Autoradiography Enhancer (Perkin Elmer cat#6NE9701) as directed by the supplier. The gel was then dried and autoradiographed at -80°C in a BioMax TranScreen LE Intensifying Screen along with Kodak “Biomax” MS film.

### Generation of Stable Cell Lines

293T cells were seeded at 3.5 × 106 cells per 10 cm plate and transfected with 10 μg of the shRNA PSR constructs together with 5 μg of the VSVg and 5 μg of the Δ8.2 helper plasmids [85] using a calcium phosphate procedure. The medium was changed 12 hours after transfection. 293T cells (2.5 × 106 cells/ml in 10 cm plates) were infected by 0.45 μm-filtered supernatent from virus-producing cells in the presence of 8 μg/ml polybrene. The cells were infected 3 times every 24 hours. After the third infection, the cells were seeded at a density of 2.5 × 106 cells per 10 cm plate and treated with 7 μg/ml of puromycin. After 3 days, the puromycin resistant cells were maintained with 5 μg/ml puromycin.

### Western-Blot Analysis

Protein lysates for western-blot were prepared in two different ways. Cells from a 10 cm plate were lysed in 500 μl of Triton lysis buffer (50 mM Tris-HCl [pH 7.5], 100 mM NaCl, 0.5 mM EDTA, 0.4% Triton, 50 mM NaF, 40 mM β-glycerophosphate, 1 mM sodium orthovanadate, 1 mM PMSF, 0.055 units/ml aprotinin). Cell extracts were collected and centrifuged at 16,000 g for 10 min. Supernatant was collected and boiled in Laemmli sample buffer (2% SDS, 10% glycerol, 5% b-mercaptoethanol, 63 mM Tris-HCl [pH 6.8], 0.0025% bromophenol blue) at 95°C for 1 min. Alternatively, to ensure chomatin and membrane bound proteins were collected, cells were lysed in RIPA buffer (50 mM Tris-HCl [pH 7.5], 150 mM NaCl, 20 mM EDTA, 1% NP-40, 0.1% SDS, 1 mM PMSF, EDTA-free protease inhibitor cocktail [Roche]). Collected extracts were sonicated for 45 s on a VirTris Virsonic Digital 600 at 6W, and then centrifuged at 16,000 g for 10 min. The supernatent was incubated at 70°C for 10 min in Laemmli sample buffer. Extracts were resolved on a by SDS PAGE (10%) and transferred to nitrocellulose membranes. The membranes were incubated with primary antibodies (K271me1, 1:500; FoxO3 NFL, 1:500; Flag, 1:2000; b-actin, 1:5000; Set9, 1:2000; H2B, 1:5000), and the primary antibodies were visualized using horseradish peroxidase-conjugated secondary antibody (Calbiochem) and ECL Western-Blot Detection Reagent (Amersham Biosciences).

### Cell Fractionation

Small-scale fractionation was carried out as described previously [86]. Briefly, the cytoplasmic fraction was collected by incubating scraped cells in Buffer A (10 mM HEPES [pH 7.9], 10 mM KCl, 1.5 mM MgCl2, 0.34 M Sucrose, 10% Glycerol, 1 mM DTT, 0.1% Triton-X-100, EDTA-free protease inhibitor cocktail [Roche]) for 10 min, and nuclei were separated by centrifugation at 1,300 g for 5 min. The nuclei were then lysed in Buffer B (3 mM EDTA, 0.2 mM EGTA, 1 mM DTT, EDTA-free protease inhibitor cocktail [Roche]) for 30 min on ice. Chromatin and other insoluble cell parts were separated from nucleoplasm fraction by centrifugation at 1,700 g for 5 min. All fractions were incubated at 70°C for 10 min in Laemmli sample buffer. The chromatin fraction was sonicated in Laemmli sample buffer for 30 s at 6W. Different fractions were analyzed by western-blot as described above.

Scoring of FoxO3 Subcellular Localization in U87 Cells U87 cells were plated in 12 well plates on glass coverslips at a density of 3 × 105 cells per well. The cells were transfected using TransIT-293 (Mirus, 4 μl per well) according to the supplier's protocol. For each transfection, 0.8 μg of FoxO3-GFP plasmid and 0.4 μg of Set9 plasmid was used. Cells were fixed 2 days post transfection with 4% paraformaldehyde (PFA) for 15 minutes. They were then washed 5 times with 1x PBS. Coverslips were mounted using Vectashield Hard Set with Dapi (Vector Laboratories) and examined under a Zeiss Axioskop2 Plus with a FLuoArc Ultraviolet module. Counting of FoxO3-GFP subcellular localization was done in a blinded manner and a minimum of 200 cells were counted per condition for each experiment. If the outline of the nucleus was clearly visible in the GFP channel, FoxO3 localization was scored as cytoplasmic. If the nucleus was clearly visible in the GFP channel, FoxO3 localization was scored as nuclear. If neither of these conditions were met, FoxO3 localization was scored as ubiquitous. Statistics were done on the percentage of cells in each condition over 3 independent experiments and significance was determined using a Bonferroni posttest.

### Immunofluorescence in NIH3T3 cells

NIH3T3 cells were plated in 6 well plates on poly-L-lysine coated glass coverslips at a density of 106 cells/well. Cells were transfected with FoxO3-GFP or FoxO3-GFP K271R and Flag-Set9 WT or H297A at a ratio of 4μg:6μg Foxo:Set9 using Lipofectamine 2000 (Invitrogen, 10 μl per well), according to the manufacturer's protocol. Cells were fixed 24 hours post-transfection with 4% PFA for 15 min. Cells were then washed 5 times with 1x PBS. Coverslips were blocked with PBS supplemented with 3% BSA and 0.1% Triton X-100 (PBS-BT) and stained with DAPI, mouse anti-GFP 3E6 monoclonal antibody (Invitrogen, 1:500), K271me1 polyclonal antibody (1:500) and appropriate secondary antibodies labeled with Alexa Fluor 488 (anti-mouse IgG) or 594 (anti-rabbit IgG), respectively (Invitrogen, 1:250). Coverslips were mounted using anti-fade mounting medium containing PBS, glycerol and P-phenylenediamine. Coverslips were imaged on an Axiovert 200M (Zeiss) equipped with a CCD camera (Hamamatsu c47429512ERG). Coverslips were imaged in a blinded manner. Contrast enhancement was performed using Photoshop CS3 (Adobe).

### Tandem Mass Spectrometry Analysis

A Coomassie-stained band corresponding to GST-FoxO3 methylated by Set9 was excised from an SDS-polyacrylamide gel, divided in half, reduced with dithiothreitol, alkylated with iodoacetamide, and digested with either trypsin or chymotrypsin. Peptide mixtures were separated by microcapillary reverse-phase chromatography and analyzed online in a hybrid linear ion trap-Orbitrap (LTQ-Orbitrap; Thermo Electron) mass spectrometer [87]. Mass spectra were data base-searched using the SEQUEST algorithm. All peptide matches were initially filtered based on enzyme specificity, mass measurement error, XCorr and DCorr scores and further manually validated for peptide identification and site localization.

### Generation of Protein Microarray, Peptide Synthesis, and Labeling

The generation of protein microarrays has been described [78]. Methylated and unmethylated forms of the K271 biotinylated peptide were synthesized by the W. M. Keck Biotechnology Resource Center (New Haven, CT). Biotinylated peptides (10 μg) were labeled as previously described [78]. A GenePix 4200A scanner (Axon, Inc.) was used for array analysis.
